# Clinical characteristics and prognosis analysis of uterine sarcoma: a single-institution retrospective study

**DOI:** 10.1186/s12885-022-10129-x

**Published:** 2022-10-07

**Authors:** Fang Wang, Xinyue Dai, Huijun Chen, Xiaoli Hu, Yuanqiu Wang

**Affiliations:** 1 Department of Pathology, First Affiliated Hospital of Wenzhou Medical University, Wenzhou, 325000 China; 2 Department of Gynecology, First Affiliated Hospital of Wenzhou Medical University, Zhejiang, Wenzhou, 325000 China

**Keywords:** Uterine sarcomas, Prognostic factors, Histological types, Tumor size

## Abstract

**Background:**

Uterine sarcomas are rare and aggressive gynaecologic malignancies, characterized by a relatively high recurrence rate and poor prognosis. The aim of this study was to investigate the clinicopathological features and explore the prognostic factors of these malignancies.

**Methods:**

This was a single-institution, retrospective study. We reviewed the medical records of 155 patients with pathologically confirmed uterine sarcomas including uterine leiomyosarcoma (ULMS), low-grade endometrial stromal sarcoma (LG-ESS), high-grade endometrial stromal sarcoma (HG-ESS), undifferentiated uterine sarcoma (UUS) and adenosarcoma (AS) between 2006 and 2022. A total of 112 patients who underwent surgery between January 2006 and April 2019 were included in the survival analysis. The current study recorded the clinicopathological, treatment and outcome data to determine clinical characteristics and survival.

**Results:**

The most common histopathological type was ULMS (63/155, 40.64%), followed by LG-ESS (56/155, 36.13%) and HG-ESS (16/155, 10.32%). The mean age at diagnosis of all patients was 49.27±48.50 years and 32.90% (51/155) of patients were postmenopausal. Fifteen patients underwent fast-frozen sectioning, 63(54.78%) were diagnosed with malignancy, 29(25.22%) were highly suspected of malignancy that needed further clarification and 23(14.84%) were diagnosed with benign disease. A total of 124(80%) patients underwent total hysterectomy (TH) and salpingo-oophorectomy. Multivariate analyses showed that histological type and tumour size were independent prognostic factors both for overall survival (OS) (*p*<0.001 and *P*=0.017, respectively) and progression-free survival (PFS) (*p*<0.001 and *P*=0.018, respectively). Tumour stage was only significantly associated with PFS (*P*=0.002). Elevated preoperative NLR, PLR and postmenopausal status were significantly correlated with shorter PFS and OS in univariate analysis, but no statistically significant difference was found in multivariate analysis.

**Conclusions:**

In patients with uterine sarcoma, in comparison to LMS and LG-ESS, UUS and HG-ESS tend to present as more aggressive tumour with poorer outcomes. Furthermore, larger tumour (>7.5 cm) were an important predictor of shorter PFS and OS.

## Introduction

Uterine sarcomas are an extremely rare and varied group of gynaecologic cancers derived from uterine mesenchymal cells, that accounts for approximately 1% of female genital tract malignancies and up to 7% of uterine malignancies [1]. Their rarity and histopathological diversity lead to a lack of consensus on the risk factors for poor prognosis and optimal treatment [2]. Uterine sarcomas are divided into categories, according to the type of cell they start in. The most common histological types are uterine leiomyosarcoma (ULMS), endometrial stromal sarcoma (ESS), undifferentiated uterine sarcoma (UUS) and adenosarcoma (AS). Endometrial stromal sarcomas were further subdivided into low-grade endometrial stromal sarcoma (LG-ESS) and high-grade endometrial stromal sarcoma (HG-ESS) by the 2014 WHO classification. Carcinosarcoma is no longer regarded as part of uterine sarcoma, but an endometrial carcinoma with sarcomatoid differentiation and therefore was treated as a high-grade endometrial carcinoma [3]. Uterine sarcomas tend to have a poorer prognosis and behave more aggressively than the more common types of endometrial cancer, not just due to the aggressiveness of the disease, but also the lack of specific symptoms, diagnostic technology and standard treatment [4]. The prognosis of uterine sarcoma is closely related to pathological type and optimal treatment [5]. ULMS, HG-ESS and UUS are associated with poor prognosis even when confined to the uterus at the time of diagnosis, whereas patients with LG-ESS and AS have later and less frequent recurrences [2]. Surgery remains the gold standard in the treatment of uterine sarcoma. The surgical approach depends on pathological type and tumour stage, and mainly consists in total hysterectomy with bilateral salpingo-oophorectomy [6]. There has been no consistency among various studies on the correlations between prognosis and patient age, clinical stage, tumour size and vascular invasion [7]. In this study, we reviewed 155 patients with uterine sarcomas diagnosed in our institution during the past 16 years, and retrospectively investigated the clinicopathological features and prognostic factors of these malignancies.

## Materials and methods

### Patients

A cohort of patients first diagnosed with uterine sarcomas who underwent surgery between January 2006 and April 2022 was collected from the First Affiliated Hospital of Whenzhou Medical University. This study was approved by the Ethical Committee of the First Affiliated Hospital of Wenzhou Medical University with the number KY2022-R098 and written informed consent was obtained. This study was a retrospective analysis of clinical data, unrelated to human bioethics. The study was performed under the principles of the Declaration of Helsinki [8]. All methods were performed under the relevant guidelines and regulations. Pathology specimens of uterine sarcoma were reviewed by the same pathologist in our hospital. Patients diagnosed with AS, ULMS, LG-ESS, HG-ESS, and UUS, according to the 2014 WHO classification system were enrolled in this study. Pre-, intra- and postoperative data were retrieved from the medical records of our hospital. Follow-up data were obtained by medical records and telephone interviews. After excluding patients with incomplete data and those lost to follow-up, a total of 155 patients were enrolled in this study. Preoperative data included patient age at the time of diagnosis, gravity, parity, body mass index (BMI), menstrual status, manifestations at visit, blood test results and ultrasonography results. Intraoperative information consisted of the surgical procedures, and frozen section diagnosis. Postoperative data included pathologic results, stage (according to International Federation of Gynecology and Obstetrics, FIGO 2009), and adjuvant treatment. A total of 112 patients with uterine sarcoma who underwent surgery between January 2006 and April 2019 were included in the survival analysis. Progression-free survival (PFS) was measured from the date of primary surgery to the date of disease recurrence or disease progression. Overall survival (OS) was defined as the time (months) from the initiation of surgery to death from any cause. The cut-off point for the survival study was April, 2022.

### Statistical analysis

Statistical analysis was conducted using IBM SPSS Statistics 20 and *P*<0.05 was considered statistically significant. Continuous variables were compared using an independent-samples t test and are presented as the means with standard deviations. Categorical variables were assessed by using Fisher’s exact test or the chi-squared test, and were expressed as numbers with percentages. The optimal cut-off values for the neutrophil/lymphocyte ratio (NLR) and platelet/lymphocyte ratio (PLR) were assessed by receiver operating characteristic (ROC) curve analysis and Youden's index. Survival curves were calculated with the Kaplan–Meier method, and any differences in survival were evaluate with a log rank test. Univariable and multivariable survival analyses were performed using Cox proportional hazards models. Prognostic factors significantly associated with PFS or OS in the univariate analysis were included in multivariate Cox regression analysis by backwards stepwise selection. Hazard ratios (HRs) estimated from the Cox-regression analysis were reported as relative risks with corresponding 95% confidence intervals (CIs).

## Results

### Preoperative characteristics

The preoperative clinical characteristics of 155 patients with uterine sarcoma are shown in Table 1. The most common histopathological type was ULMS (63/155, 40.64%), followed by LG-ESS (56/155, 36.13%) and HG-ESS (16/155, 10.32%), and UUS is the rarest in our study, accounting for 3.23% (5/155). Most patients had stage 1 disease (130/155, 83.87%), 12 (7.74%) patients had stage 2 disease, 5(3.22%) had stage 3 disease and 8 (5.16%) patients had stage 4 disease. Of all these patients, only 31(20%) were suspected to have malignancies before surgery by ultrasound. Approximately 80% of uterine sarcomas are misdiagnosed preoperatively. The mean age at diagnosis of all patients was 49.27±48.50 years and 51(32.90%) patients were postmenopausal. As mentioned in the literature [9], the incidence rate varies according to age. Women with UUS, compared with those with other pathological types, tended to be older, of which 4/5(80%) were postmenopausal. The mean tumour size was 58.07±29.33 mm for AS, 80.16±36.65 mm for ULMS, 66.14±30.13 mm for LG-ESS, 61.19±35.31 mm for HG-ESS, and 94.8±47.96 mm for UUS, which was the largest. The primary presenting complaints in patients with uterine sarcoma included pelvic mass, abnormal uterine bleeding (AUB), postmenopausal bleeding (PMB) and abdominal/pelvic pain. Except ULMS and UUS, the most common manifestation on examination was AUB. Intramural tumours were predominant in our study, while women with AS and UUS were more likely to have tumours that were subserosal, and were prone to having solitary masses. The preoperative serum markers CA-125, CA1-99 and LDH have limited value in the diagnosis of uterine sarcoma; abnormally elevated values occurred in less than 30% of patients.

**Table 1:** Preoperative clinical characteristics of 155 patients with uterine sarcoma

Parameters	**AS** ***N*=15**	**ULMS** ***N*=63**	**LG-ESS** ***N*=56**	**HG-ESS** ***N*=16**	**UUS** ***N*=5**	**Total** ***N*=155**
**Age (y)**	51.73±11.91	50.43±8.23	45.73±10.77	49.75±8.36	62.40±1.48	49.27±48.50
**Gravidity**	3.40±1.72	3.25±1.48	3.46±1.67	3.44±2.06	4.00±1.22	3.36±3.00
**Parity**	2.00±1.31	2.06±0.95	1.80±0.98	1.81±0.91	2.80±1.48	2.03±2.00
**Size(mm)**	58.07±29.33	80.16±36.65	66.14±30.13	61.19±35.31	94.8±47.96	69.09±66.50
**BMI (kg/m2)**	23.91±3.93	23.39±3.22	22.89±2.80	22.87±3.02	24.05±2.34	23.40±23.44
**Localization**						
**Intramural**	2(13.33)	30(47.62)	33(58.93)	9(56.25)	2(40)	76(49.03)
**Subserous**	2(13.33)	16(25.40)	4(7.14)	1(6.25)	1(20)	24(15.48)
**Submucous**	11(73.33)	17(26.98)	19(35.83)	6(37.50)	2(40)	55(34.48)
**Number of masses**						
**1**	13(86.67)	42(66.67)	27(48.21)	6(37.50)	4(80%)	92(59.35)
**≥2**	2(13.33)	21(33.33)	29(51.79)	10(62.50)	1(20%)	63(40p.65)
**Menopause**						
**Pre-menopausal**	8(53.33)	39(61.90)	47(83.93)	9(56.25)	1(20%)	104(67.10)
**Postmenopausal**	7(46.67)	24(38.10)	9(16.07)	7(43.75)	4(80%)	51(32.90)
**Manifestations**						
**Pelvic Mass**	1(6.67)	23(36.51)	15(26.79)	2(12.50)	2(40%)	43(27.74)
**AUB**	6(40.00)	25(39.68)	23(41.07)	7(43.75)	0(0)	61(39.35)
**PMB**	7(46.67)	8(12.70)	5(8.93)	5(31.25)	3(60%	28(18.06)
**Pain**	1(6.67)	7(11.11)	13(23.21)	2(12.50)	0(0)	23(14.84)
**serum marker**						
**CA-125 abnormality**	3/12(25.00)	12/58(20.69)	10/51(19.61)	3/13(23.08)	0/5(0)	28/139(20.14)
**CA19-9 abnormality**	2/12(16.67)	2/58(3.45)	2/49(4.08)	1/14(7.14)	0/5(0)	7/138(5.07)
**LDH abnormality**	2/5(40.00)	12/33(36.36)	6/27(22.22)	1/6(16.67)	1/3(33.33)	22/74(29.73)
**US**						
**Benign**	10(66.67)	47(74.60)	30(53.57)	9(56.25)	3(60.00)	99(63.87)
**Degeneration**	0(0)	7(11.11)	16(28.57)	1(6.25)	1(20.00)	25(16.13)
**Malignant tumors**	5(33.33)	9(14.29)	10(17.86)	6(37.50)	1(20.00)	31(20.00)

*Abbreviations*: *BMI* Body mass index, *AUB* Abnormal uterine bleeding, *PMB* Postmenopausal bleeding

### Intraoperative and postoperative diagnosis and treatment

A total of 39 patients underwent myomectomy as the initial surgery. Of these patients, 33(84.62%) patients underwent secondary staging operations with total hysterectomy, while 6(15.38%) patients did not undergo supplementary surgery. Finally, patients who underwent total hysterectomy (TH) and salpingo-oophorectomy, which were the most common surgical methods, accounted for 80%. Sixty-nine (44.52%) patients underwent lymph node dissection. However, in the ULMS group, only 27% of patients underwent lymph node dissection. Five patients underwent unilateral salpingo-oophorectomy (UBO) because they were premenopausal at the time of surgery. More than half of the patients (84/155, 54.19%) underwent laparotomy as the initial surgery and 71(45.81%) patients underwent laparoscopy. Nineteen (12.26%) patients in our study underwent preoperative endometrial sampling or transvaginal neoplasm biopsy, and 16 were diagnosed with uterine malignancy. Of the 115 patients (including 10 patients who underwent preoperative endometrial sampling) who underwent fast-frozen sectioning, 63(54.78%) were diagnosed with uterine malignancy, 29(25.22%) were highly suspected of malignancy that needed further clarification and the remaining 23(14.84%) patients were diagnosed with benign disease. More than four-fifths of the patients (130 patients, 83.37%) were diagnosed as stage I. Approximately two-fifths (64 patients, 43.58%) of the patients received adjuvant chemotherapy, and two of them received radiotherapy at the same time. The pathological results and treatments for patients with uterine sarcoma are shown in Table 2.

**Table 2:** Pathological results and treatments for patients with uterine sarcoma

**parameters**	**AS** ***N*=15**	**ULMS** ***N*=63**	**LG-ESS** ***N*=56**	**HG-ESS** ***N*=16**	**UUS** ***N*=5**	**Total** ***N*=155**
**Surgical procedures**						
**Myomectomy**	1(6.67)	4(6.35)	1(1.79)	0(0)	0(0)	6(3.87)
**TH**	0(0)	10(15.87)	9(16.07)	0(0)	0(0)	19(12.26)
**H+USO**	0(0)	0(0)	0(0)	1(6.25)	0(0)	1(0.65)
**H+BSO**	5(33.33)	32(50.79)	16(28.57)	6(37.50)	1(20.00)	60(38.71)
**H+BSO+PLND**	8(53.33)	16(25.40)	27(48.21)	9(56.25)	4(80.00)	64(41.29)
**H+USO+ PLND**	1(6.67)	1(1.59)	3(5.36)	0(0)	0(0)	5(3.23)
**Mode of initial surgery**						
**Laparoscopy**	4(26.67)	32(50.79)	29(51.79)	5(31.25)	1(20.00)	71(45.81)
**Laparotomy**	11(73.33)	31(49.21)	27(48.21)	11(68.75)	4(80.00)	84(54.19)
**frozen section**						
**Malignant**	6/9(66.67)	24/51(47.06)	19/40(47.50)	9/10(90.00)	5/5(100)	63/115(54.78)
**Malignant suspected**	1/9(11.11)	20/51(39.22)	7/40(17.50)	1/10(10.00)	0/5(0)	29/115(25.22)
**Begin**	2/9(22.22)	7/51(13.72)	14/40(35.00)	0/10(0)	0/5(0)	23/115(14.84)
**FIGOa**						
**I**	11(73.33)	55(87.30)	47(83.93)	13(81.25)	4(80.00)	130(83.87)
**II**	1(6.67)	4(6.35)	5(8.93)	1(6.25)	1(20.00)	12(7.74)
**III**	0(0)	3(4.76)	2(3.57)	0(0)	0(0)	5(3.22)
**IV**	3(20.00)	1(1.59)	2(3.57)	2(12.50)	0(0)	8(5.16)
**Adjuvant treatment**						
**chemotherapy**	5(33.33)	33(52.38)	13(23.21)	10(62.50)	3(60.00)	64(41.29)
**radiotherapy**	0(0)	1(1.59)	0(0)	0(0)	0(0)	1(0.65)
**Both**	0(0)	1 (1.59)	0(0)	1(6.25)	0(0)	2(1.29)
**None**	10(66.67)	28(44.44)	43(76.79)	5(31.25)	2(40.00)	88(56.77)

*Abbreviations*: *TH* Total hysterectomy, *USO* Unilateral salpingo-oophorectomy, *BSO* Bilateral salping-oophorectomy, *PLND* Pelvic lymphadenectomy

### Relationship between clinicopathologic parameters and survival

**Fig. 1:**
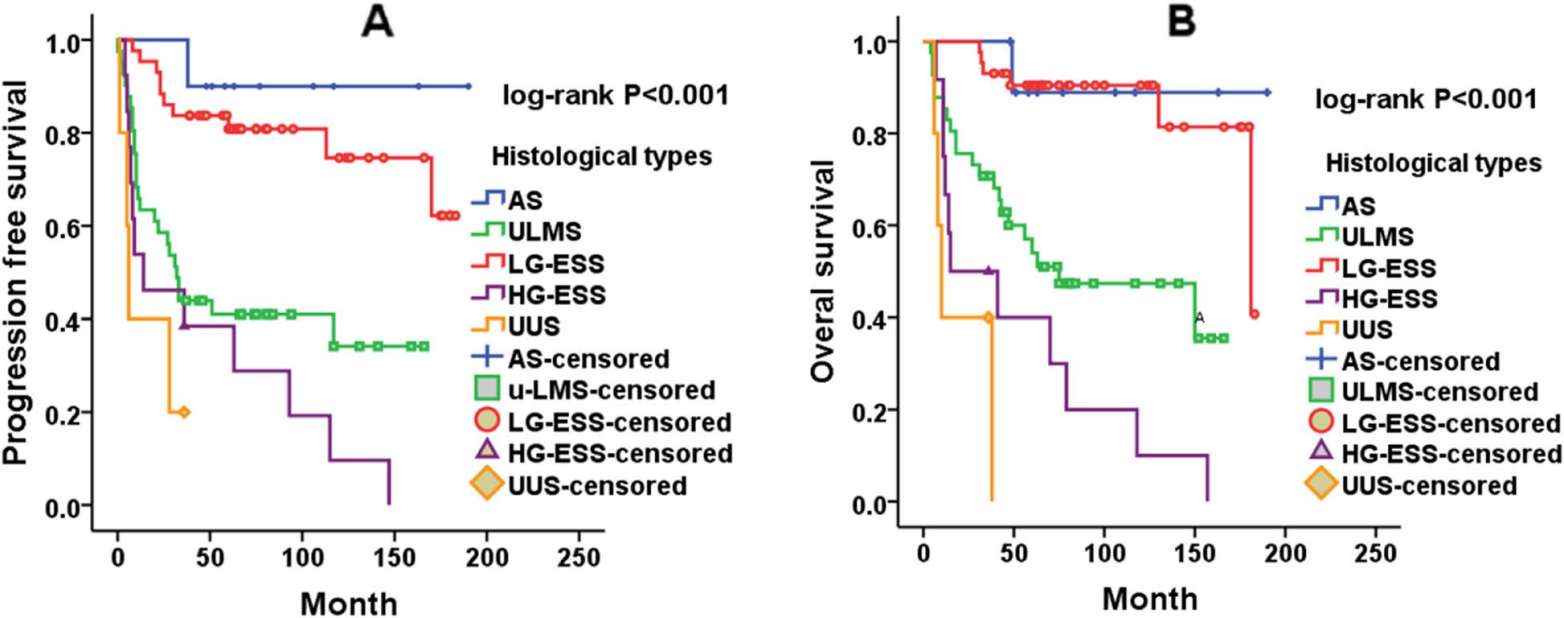
Kaplan–Meier survival curves of tumour histological types for PFS (**A**) and OS (**B**)

**Fig. 2:**
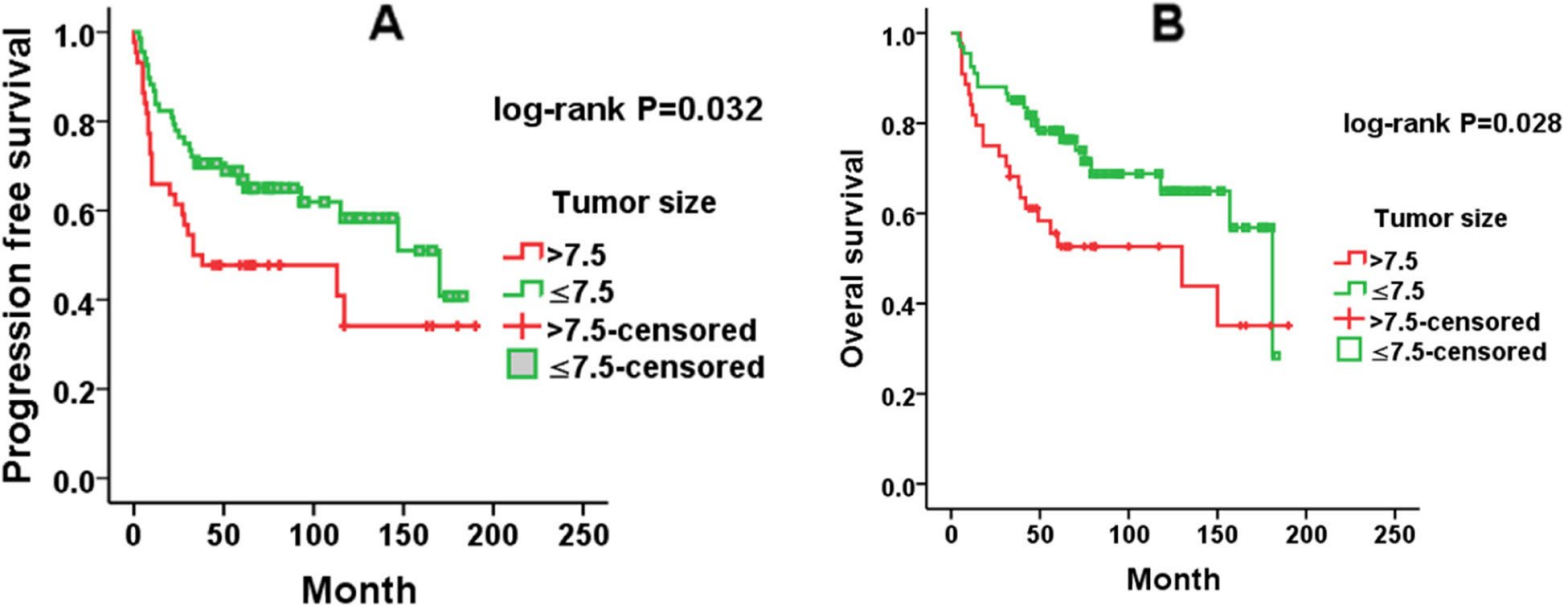
Kaplan–Meier survival curves of tumour size for PFS (**A**) and OS (**B**)

**Fig. 3:**
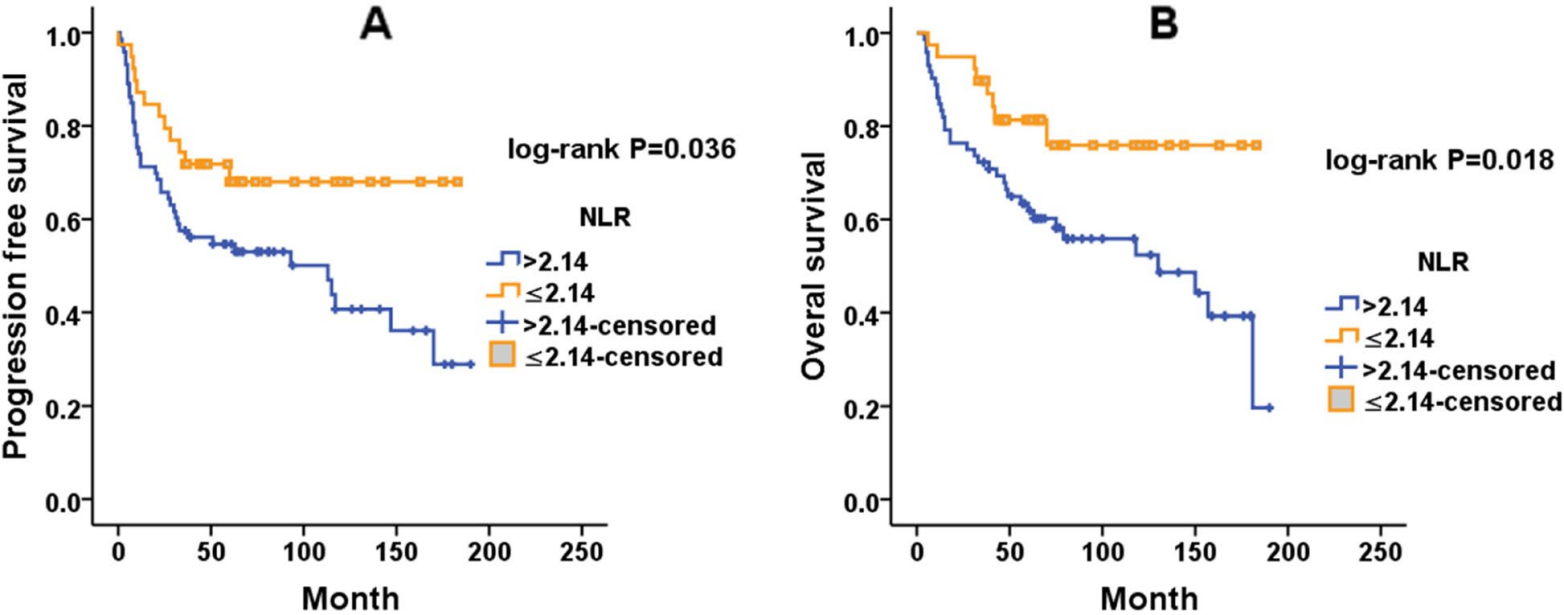
Kaplan–Meier survival curves of preoperative NLR for PFS (**A**) and OS (**B**)

A total of 112 uterine sarcoma patients who underwent surgery between January 2006 and April 2019 were enrolled in the survival analysis. The median progression-free survival(PFS)and overall survival (OS) times were 54 months (range 1-190 months) and 63.5 months (range 4-190 months), respectively. The estimated cumulative 5-year survival rates for this population were 54.9 ± 4.7% for PFS and 68.2 ± 4.5% for OS. The optimal cut-off values were 2.14 for NLR and 128.5 for PLR, according to ROC analysis. Univariate analysis suggested that tumour histological type (*P*<0.001), FIGO stage (I vs. II–IV: hazard ratio = 2.771, *P* = 0.003), tumour size (hazard ratio = 1.796, *P* = 0.035), menstrual status (hazard ratio = 2.264, *P* = 0.004), preoperative NLR (hazard ratio =1.973, *P* = 0.039) and PLR (hazard ratio = 2.514, *P* = 0.017) were all significantly associated with PFS, whereas age at diagnosis, lymphadenectomy, ovariectomy and the mode of initial surgery were not (Table 3). We found significant associations between tumour histological type (*P*<0.001), tumour size (hazard ratio =1.932, *P* = 0.031), menstrual status (hazard ratio =1.956, *P* = 0.032), NLR (hazard ratio =2.451, *P* = 0.022), and PLR (hazard ratio =3.459, *P* = 0.009) and OS (Table 4). Factors with significant difference in the univariate analysis were included in multivariate Cox regression analysis. The multivariate analysis results indicated that tumour histological type, FIGO stage, and tumour size were all independent prognostic factors for PFS (*P*<0.001, *P*=0.002 and *P*=0.018, respectively) (Table 3). The above indicators, except FIGO stage were also significantly associated with OS (all *P*<0.05) (Table 4). As shown in Fig. 1, tumour histological type was significantly correlated with PFS and OS (both log rank *P*<0.001). Patients with AS and LG-ESS tended to have better PFS and OS, and patients with UUS had the worst PFS and OS. Kaplan–Meier analysis (Fig. 2) indicated that tumour size>7.5 cm had significantly shorter PFS (*P*=0.032) and OS (*P*=0.028). Although menstrual status**,** NLR and PLR were not significant in multivariate analysis, they were significantly correlated with PFS and OS in univariate analysis. An NLR ≤2.14 was associated with better PFS (*P*=0.035) and OS (*P*=0.018) according to Kaplan–Meier analysis (Fig. 3).

**Table 3:** Univariate and multivariate Cox proportional hazards regression analyses for progression free survival of uterine sarcoma patients (*n* =112)

Parameter	Univariate analysis		Multivariate analysis	
	HR (95%CI)	*P*-value	HR (95%CI)	*P*-value
Histological types		<0.001		<0.001
AS(*N*=10)	1 (referent)		1 (referent)	
ULMS(*N*=41)	14.062(1.623-121.837)	0.016	12.848(1.711-96.487)	0.013
LG-ESS(*N*=43)	2.727(0.307-24,217)	0.368	3.476(0.438-27.594)	0.239
HG-ESS(*N*=13)	108.000(5.922-1969.539)	0.002	24.223(3.039-193.068)	0.003
UUS(*N*=5)	36.000(1.772-731.562)	0.020	40.268(4.307-376.520)	0.001
FIGO stage				
Stage I	1 (referent)		1 (referent)	
Stage II-IV	3.300(1.077-10.114)	0.037	3.031(1.524-6.029)	0.002
Lymphadenectomy				
Yes	1 (referent)			
No	0.939(0.444-1.986)	0.869		
Initial surgery				
Laparotomy	1 (referent)			
Laparoscopy	1.604(0.746-3.451)	0.227		
Ovariectomy				
Yes	1 (referent)			
No	1.375(0.531-3.562)	0.512		
Age(years)				
≤50	1 (referent)			
>50	1.852 (10.862-7.255)	0.114		
Size				
≤7.5	1 (referent)		1 (referent)	
>7.5	1.998(0.926-4.312)	0.078	2.100(1.136-3.881)	0.018
Menstrual status				
Premenopausal	1 (referent)			
Postmenopausal	2.743 (1.199-6.272)	0.017		
NLR				
≤2.14	1 (referent)		1 (referent)	
>2.14	2.7271(1.199-6.201)	0.017	1.884(0.938-3.625)	0.076
PLR				
≤128.5	1 (referent)			
>128.5	3.419 (1.369-8.541)	0.009		

*Abbreviation:* *AS* Adenosarcoma, *ULMS* Uterine leiomyosarcoma, *LG-ESS* Low-grade endometrial stromal sarcoma; *HG-ESS* High-grade endometrial stromal sarcoma, *UUS* Undifferentiated uterine sarcoma, *NLR* Neutrophil to lymphocyte ratio, *PLR* Platelet to lymphocyte ratio, *HR* Hazard ratio, *CI* Confidence interval

**Table 4:** Univariate and multivariate Cox proportional hazards regression analyses for overall survival of uterine sarcoma patients (*n* =112)

Parameter	Univariate analysis		Multivariate analysis	
	HR (95%CI)	*P*-value	HR (95%CI	*P*-value
Histological types		<0.001		<0.001
AS(*N*=10)	1 (referent)		1 (referent)	
ULMS(*N*=41)	9.450 (1.095-81.920)	0.041	10.485(1.170-93.928)	0.036
LG-ESS(*N*=43)	1.459(0.156-13.693)	0.741	2.371(0.255-22.050)	0.448
HG-ESS(*N*=13)	99.000(5402-1814.474)	0.002	38.355(3.881-379.020)	0.002
UUS(*N*=5)	36.000(1.772-731.562)	0.020	47.622(4.315-525.554)	0.002
FIGO stage				
Stage I	1 (referent)			
Stage II-IV	1.498(0.530-4.240)	0.446		
Lymphadenectomy				
Yes	1 (referent)			
No	1.619(0.620-4.227)	0.852		
Initial surgery				
Laparotomy	1 (referent)			
Laparoscopy	1.508(0.684-3.327)	0.309		
Ovariectomy				
Yes	1 (referent)			
No	0.632(0.233-1.713)	0.325		
Age(years)				
≤50	1 (referent)			
>50	1.496(0.688-3.250)	0.309		
Size				
≤7.5	1 (referent)		1 (referent)	
>7.5	2.190(0.999-4.801)	0.050	2.277(1.157-4.484)	0.017
Menstrual status				
Premenopausal	1 (referent)			
Postmenopausal	2.250 (0.990-5.116)	0.053		
NLR				
≤2.14	1 (referent)		1 (referent)	
>2.14	3.666(1.484-9.054)	0.005	2.026(0.919-4.468)	0.080
PLR				
≤128.5	1 (referent)			
>128.5	4.705(1.642-13.484)	0.004		

*Abbreviations*: *AS* Adenosarcoma, *ULMS* Uterine leiomyosarcoma, *LG-ESS* Low-grade endometrial stromal sarcoma, *HG-ESS* High-grade endometrial stromal sarcoma, *UUS* Undifferentiated uterine sarcoma, *NLR* Neutrophil to lymphocyte ratio, *PLR* Platelet to lymphocyte ratio, *HR* Hazard ratio, *CI* Confidence interval

## Discussion

Large sample studies of uterine sarcomas are difficult to carry out in a single institution because they are rare gynaecological tumours with diverse histological features. Our retrospective study over an 18-year period provided insights into clinicopathological features of 155 patients and explored the prognostic factors of 112 patients diagnosed with uterine sarcomas. These tumours are characterized by a relatively high recurrence rate and poor prognosis. Preoperative diagnosis of uterine sarcoma is usually difficult and frequently not possible. Its diagnostic accuracy is not satisfactory. Patients are often treated for presumed uterine leiomyomas and diagnosed incidentally on hysterectomy specimen analysis [10, 11]. Although magnetic resonance imaging (MRI) has a developing role in the assessment of these malignancies [12], it is unlikely to become a universal preoperative evaluation method because of its high price. The initial evaluation of all our patients was performed using pelvic ultrasound (US). As shown in Table 1, 63.87% of cases were misdiagnosed as uterine leiomyoma and 16.13% were regarded as degenerative myoma by preoperative US. The mainstay for the preoperative diagnosis of uterine malignancy is endometrial sampling [13]. Twenty-five percent of cases were determined following endometrial sampling and 65% were diagnosed with a uterine sarcoma preoperatively, as reported by a multicentre, retrospective study of 302 patients [14]. However, uterine sarcoma is not easily diagnosed by preoperative endometrial sampling because sarcomas originate in the deep muscular myometrial layer of the uterus [15]. In our study, 8/10 patients who underwent preoperative endometrial sampling and 9/9 patients who underwent transvaginal neoplasm biopsy were diagnosed preoperatively with a uterine malignancy. Among the 17 patients diagnosed with uterine malignancy before surgery, 9 patients had AS, 10 patients had endometrial stromal tumours (LG-ESS and HG-ESS) and one patient had ULMS. Most of the sampling in our study was due to abnormal uterine bleeding. Endometrial sampling in patients presenting with abnormal uterine bleeding may exclude uterine leiomyoma, and assist surgeons in customizing the necessary surgical scope for patients.

ULMS was the most common histopathological subtype in our institution, accounting for 40.64% of uterine sarcoma cases, which is in accordance with that in previous publications [3, 10, 16, 17], describing a range between 40% and 60% of uterine sarcomas. However, a study of 114 patients performed at four affiliated hospitals of medical colleges in western China demonstrated that LG-ESS was the most frequently reported (43.9%), followed by LMS in 29.8%, and HG-ESS in 11.4% [5]. Similar to this study, the age at presentation varies with the different histologic subtypes. Patients in the LG-ESS and ULMS groups were younger, the great majority of whom were premenopausal at diagnosis, while patients with UUS had the oldest average age. In our institution, the mean age for ULMS was 50 years, and that for LG-ESS was 45 years, whereas for UUS, the mean age was 62 years. However, our analysis shows no difference for OS and PFS, contrary to the study published by Hosh et al. [18]. They analysed 13089 patients from the SEER database and found that women aged 50 years or older had worse 5-year relative survival.

Total hysterectomy represents the standard treatment for uterine sarcomas. Whether bilateral salpingo oophorectomy (BSO) should be performed remains controversial. Ovarian preservation does not adversely affect the survival in premenopausal patients with uterine leiomyosarcoma, which has been confirmed by many studies [19–21]. They insisted that BSO is not required for premenopausal women with ULMS, unless the ovary is macroscopically involved. This finding is different from other series, which reported that BOS is associated with improved prognosis in ULMS [22].

ESS is known as a hormone responsive tumour and bilateral adnexectomy is recommended due to a higher risk of recurrence in women with preserved ovarian function [2, 23].

However, other retrospective studies evaluated the safety of ovarian preservation and reached conflicting conclusions. A study of 348 patients found that ovarian preservation had no effect on overall survival in LG-ESS [24]. Similarly, some scholars began to promote the consideration of preserving ovarian function in premenopausal women with LG-ESS, especially in the early stage [25, 26], but further studies with larger group of patients are needed to confirm these findings. In our study, patients who underwent BSO had no significant difference in PFS or OS compared with patients who did not. Our conclusion is supported by Nasioudis et al. [27] , who proposed that ovarian preservation was not associated with worse survival in premenopausal women with stage I uterine sarcoma. The prognostic significance of lymphadenectomy in uterine sarcoma remains unclear. Shah et al. [24] reported that 26% ((100 of 383) of LG-ESS patients underwent lymphadenectomy and the incidence of lymph node metastasis was 7% (7 of 100). They found that lymph node metastasis had no statistically significant effect on survival. Nasioudis et al. [28] recently published a study of 6412 patients evaluating the role of lymphadenectomy for apparent early stage uterine sarcoma. It was found in this report that the incidence of lymph node metastasis was low and that lymphadenectomy was not associated with better survival in patients with AS, LG-ESS or ULMS while patients with HG-ESS and UUS who underwent lymphadenectomy had better survival. No significant difference was found in PFS or OS for lymphadenectomy in our research. We also found that there was no statistically significant difference in survival among patients with any of the tumour types who underwent laparoscopic surgery compared with those who underwent laparotomy.

Various prognostic factors have been well recognized from previous retrospective data. Consistent with previous publications [16, 29, 30], we found a significant difference in prognosis with histologic type in both univariate and multivariate analyses. AS and LG-ESS presented as less aggressive diseases with favourable outcomes, with 5-year overall survival rate above 85%, HG-ESS and UUS had an adverse prognosis with 5-year overall survival rate below 40%. Pathologists have suggested abandoning the classification of uterine sarcomas as “high-grade endometrial stromal sarcoma” because of the aggressive behaviour and poor outcomes [30]. UUS and HG-ESS have been reclassified by the World Health Organization (WHO) as high-grade uterine sarcomas [16]. The overall 5-year survival rate for patients with ULMS was 54%, which is agreement with the findings in a previous study that estimated 25–76% for local disease and 10–15% for metastatic disease [31]. Tumour size has been reported to be of prognostic value in uterine sarcoma. D’Angelo et al. published a retrospective study examining the effect of clinicopathologic parameters on survival outcomes in ULMS [32]. They included two medical centres from 1978 to 2008 with a total of eighty-four ULMS patients. They proposed a large tumour size (> 100 mm) was associated with a poorer prognosis. A single-institutional study also showed that tumours greater than 10 cm were an important predictor for disease-free survival [33]. This is in accordance with present study, while the cut-off value of tumour diameter in this study was 7.5 cm. Tumour stage was also considered as an important prognostic factor for uterine sarcoma, with 5-year overall survival being 50–55% for stage I disease and 8–12% for stage II–IV disease.

Li et al. [5] observed a significant association between tumour stage and overall survival in both univariate and multivariate analyses. The 3-year OS for all histological types was 75.4% for stage I, 57.1% for stage II, 33.3% for stage III and 7.1% for stage IV. This association is similar to the previous publication in ULMS and ESS [34, 35]. In our study, early stage (stage I) was associated with better PFS than advanced stage (stage II-IV), but there was no difference in OS. Although we did not find a difference in OS, this could be attributed to the following reasons. First, the number of patients in the advanced stage group was small, therefore, the study did not have the number of events required to demonstrate a significant difference between the two groups. Second, as a more aggressive uterine sarcoma, more than 80% of patients with HG-ESS and UUS were in stage I at diagnosis. Considering the association between histopathological subtype and poor prognosis, it is difficult to determine whether advanced stage has any effect on the poor outcome.

In our study, elevated preoperative NLR and PLR and postmenopausal status were significantly correlated with shorter PFS and OS in univariate analysis, but no statistically significant difference was found in multivariate analysis. The NLR was more meaningful in multivariate analysis than the other two indicators (*P*<0.1). Recently, Jeong et al. [36] performed a retrospective analysis of 99 patients with uterine sarcoma from 8 multicentre institutions over the last 20 years and found that a high NLR was significantly associated with worse DFS and OS, while PLR, CA125, and lactate dehydrogenase (LDH) failed to show significant impact.

In conclusion, uterine sarcoma is a rare tumour, with ULMS being the most frequent (40.64%), followed by LG-ESS (36.13%) and HG-ESS (10.32%). UUS and HG-ESS are more aggressive. Our study illustrated that histologic type, tumour size and tumour stage for patients with uterine sarcoma may act as independent predictors of PFS, as well as OS except tumour stage.

## Data Availability

The datasets used in the current study are available from the corresponding author upon reasonable request.
